# Interleukin-8 in health and disease

**DOI:** 10.1186/s43556-026-00511-7

**Published:** 2026-07-09

**Authors:** Chiara Bazzichetto, Emanuela Dell’Aquila, Margherita Veroli, Lorenzo Belluomini, Adele Bonato, Giovanni Bernardini, Michele Bevilacqua, Filippo Cattazzo, Andrea Dalbeni, Tarcisio Michele Tampu, Valentina Salari, Paolo Francesco Fabene, Claudio Luchini, Michele Milella, Fabiana Conciatori

**Affiliations:** 1 Preclinical Models and New Therapeutic Agents Unit, IRCCS - Regina Elena National Cancer Institute, Rome, Italy; 2 Division of Medical Oncology 2, IRCCS - Regina Elena National Cancer Institute, Rome, Italy; 3 Section of Innovation Biomedicine - Oncology Area, Department of Engineering for Innovation Medicine (DIMI), University of Verona, and Verona University and Hospital Trust (AOUI), Verona, Italy; 4 Department of Molecular Medicine, Sapienza University of Rome, Rome, Italy; 5 Section of General Medicine C and Liver Unit, University and Azienda Ospedaliera, Universitaria Integrata of Verona, Verona, Italy; 6 Section of Innovation Biomedicine - Department of Engineering for Innovation Medicine (DIMI), University of Verona, Verona, Italy; 7 FutureNeuro Research Ireland Centre for Translational Brain Science, RCSI University of Medicine and Health Sciences, Dublin, D02 YN77 Ireland; 8 Section of Anatomy and Histology - Department of Excellence in Neurosciences, Biomedicine and Movement Sciences, University of Verona, Verona, Italy; 9 Section of Pathology, Department of Diagnostics and Public Health - ARC-Net Research Center, University of Verona, Verona, Italy

**Keywords:** Interleukin-8, Immune system, Biomarker, Non-malignant diseases, Cancer

## Abstract

Cytokines are among the main factors involved in indirect cell-to-cell communication in physiological and pathological conditions. In this way, cytokines are soluble and tissue-resident easily detectable biomarkers for the diagnosis and the prediction of clinical outcome, as well as drug targets. Over the years, the pleiotropic IL-8 gained a central role in the pathogenesis of several immunological disorders, spanning from autoimmune pathologies and sepsis to neurological, liver and cardiovascular diseases. In several solid cancers, IL-8 not only serves as a prognostic biomarker but also as predictive tool. Increasing evidence highlighted IL-8 as a prognostic/predictive biomarker even for immunotherapy to stratify eligible patients, to avoid unnecessary toxicity and progression. Today, a comprehensive review covering IL-8 in all these fields has not yet been published. Hence, we aim to provide preclinical and clinical evidence on the biology and functions of IL-8, involved in the pathogenesis of non-malignant diseases, including inflammatory bowel diseases, sepsis, neuroinflammation, liver and cardiovascular pathologies. In oncology area, we will describe the pro-tumorigenic roles, including the characterization of the immunosuppressive microenvironment, also discussing the implications of direct/indirect IL-8 targeting and the involvement of gut microbiota. Unfortunately, the main obstacles to establish IL-8 as a useful non-invasive clinical tool for diagnosis and treatments’ selection are the lack of the standardization of method detection and the source of IL-8 production.

## Introduction

Most intercellular communication is mediated by cytokines, which act as hormone‑like peptides. These molecules participate in virtually all physiological processes and in every domain of pathology and clinical medicine, where they also serve as biomarkers and therapeutic targets. Produced by numerous cell types, cytokines are central components of immune system networks, and fluctuations in their concentrations in tissues and biological fluids can act as sensitive indicators of homeostatic balance [1]. While temporally coordinated and appropriately scaled changes in their levels are essential to maintain physiological homeostasis, sudden and excessive increases in cytokine release can cause organ damage and even death. For instance, the poor prognosis of some patients with Coronavirus Disease 2019 (COVID‑19) has been attributed to a “cytokine storm syndrome” [2–4].

Based on a mix of primary biological function, source cell type, and chemotactic activity/receptor system, cytokines are commonly classified into tumor necrosis factor (TNF), interleukin (IL), chemokines, lymphokines, monokines, interferon (IFN), colony‑stimulating factors, and transforming growth factor (TGF). Chemokines are small chemoattractant cytokines involved in embryonic development, innate and adaptive immunity, wound healing, and angiogenesis, as well as in chronic inflammation, tumorigenesis, and autoimmune diseases [5–7]. The contribution of cytokines to cancer cell growth, tumor‑promoting inflammation, and immune evasion is well established [8–10]. Several cytokines have been extensively investigated as prognostic or predictive biomarkers and as potential therapeutic targets in malignant diseases, particularly with regard to primary resistance to immune checkpoint inhibitors (ICI) in cancer treatment [11–15].

IL‑8 was initially characterized as a chemoattractant for C-X-C motif chemokine receptor (CXCR)1/2‑positive polymorphonuclear neutrophils (PMN) and is now recognized as a key soluble mediator in several severe immunological, cardiovascular, and neurological diseases, including acute respiratory distress syndrome, Alzheimer’s disease, depressive disorders, and atherosclerosis [16–20].

In this comprehensive Review, we aim to summarize the biological and therapeutic roles of IL‑8 in health and disease, including sepsis, autoimmune conditions, liver and cardiovascular disorders, neuroinflammation, and cancer. A particular attention to its impact on immune status and, consequently, on the outcomes of immunotherapy in solid tumors will be highlighted.

## The molecular and cellular biology of IL-8

### Genomic organization and transcriptional regulation

**Fig. 1:**
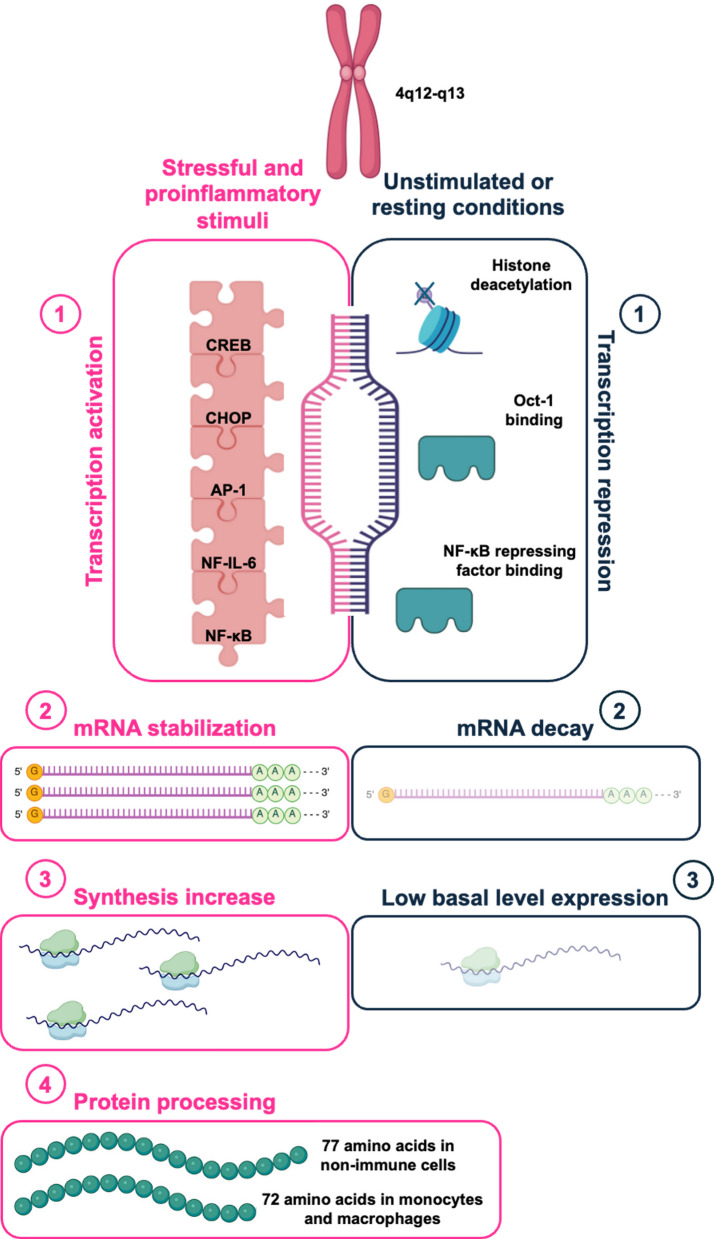
Regulation of IL-8 expression in normal and cancer tissues. IL-8 production is mainly regulated by transcriptional activation and mRNA stabilization. Left panels: upon stressful and proinflammatory stimuli, several transcription factors enhance *IL-8* transcription (1), mRNA is stabilized (2), IL‑8 synthesis increases (3) and protein is lastly processing (4). Right panels: under unstimulated or resting conditions, the *IL-8* gene is transcriptionally repressed (1), mRNA decays (2) and ow basal level of expression is guaranteed (3)

The *IL-8* gene is located at 4q12-q13. *IL-8* polymorphisms include a total of 734 mapped single nucleotide polymorphisms (SNP), occurring in both coding and non-coding regions. Several of these non‑coding *IL-8* variants, including − 251 T/A (rs4073), + 781 C/T (rs2227306), + 1633 C/T (rs2227543) and + 2767 A/T (rs1126647), have been associated with altered IL‑8 expression and with susceptibility or outcome in inflammatory, infectious, and neoplastic diseases. However, their functional impact appears to be largely regulatory and context‑dependent, and results are not fully consistent across studies [21]. IL-8 production is mainly regulated by transcriptional activation and mRNA stabilization (Fig. 1). Gene promoter contains binding regions for the following transcription factors: cAMP response element binding protein (CREB), C/EBP homologous protein (CHOP), activating protein-1 (AP-1), CAAT/enhancer-binding protein (C/EBPβ, also known as NF-IL-6), nuclear factor-κB (NF-κB) [22]. Under unstimulated or resting conditions, the *IL-8* gene is transcriptionally repressed by histone deacetylation, octamer (Oct)−1 binding, and active repression by NF-κB repressing factor. In the context of colorectal cancer (CRC), our group has recently demonstrated that IL-8 production is tightly controlled by the MAPK cascade through CHOP-dependent transcriptional activation, a regulatory mechanism alternative or complementary to the canonical control mediated by NF-κB. This, in turn results in very high level of IL-8 secretion by CRC cells carrying *BRAF* mutations and PTEN loss [23]. Hwang et al*.* also demonstrated that Snail overexpression induces IL-8 production in CRC cells, by directly binding two specific E-boxes in the *IL-8* promoter [24]. mRNA stability is another important mechanism regulating IL-8 expression: the AU-rich elements in the 3′-untranslated regions guarantee low basal level of expression without stimulation. Upon stressful and proinflammatory stimuli (such as TNF‑α and IL‑1), influence mRNA decay *vs* stabilization in response to p38 MAPK activation occurs. Stabilization of IL-8 mRNA prolongs its half‑life and increases IL‑8 synthesis without changing transcription rate. Some of these mechanisms involved in IL-8 mRNA stability differ according to the type of cells (e.g., macrophages) [25, 26]. At the protein level, IL‑8 can be further modulated by processing, secretion, and degradation, although protein turnover contributes less prominently to the regulation of IL-8 levels. At the end of protein translation, an IL-8 precursor consisting of 99 amino acids is produced and then processed into two active, cell-type specific, mature isoforms consisting of 72 amino acids in monocytes and macrophages and 77 amino acids in non-immune cells [27].

### Protein structure, receptors and downstream pathways

IL-8 protein structure was determined by nuclear magnetic resonance spectroscopy in 1990 [28]. As a cysteine-X-cysteine chemokine ligand (CXCL), IL-8 has two pairs of cysteines forming disulfide bonds (i.e., Cys-7 and Cys-34; Cys-9 and Cys-50) that are essential for structural integrity. IL-8 reversibly exists as soluble monomer and dimer. Starting from the N-terminal residues of the monomer, the protein has one turn of a 3_10_-helix, followed by three β-strands and a C-terminal α-helix [28, 29]. The three-dimensional structure of the IL-8 dimer consists of two anti-parallel α-helices (24 Å length, 14 Å distance), top on a six-stranded antiparallel β-sheet [28]. The total structure weight is 8-12 kDa.

**Fig. 2:**
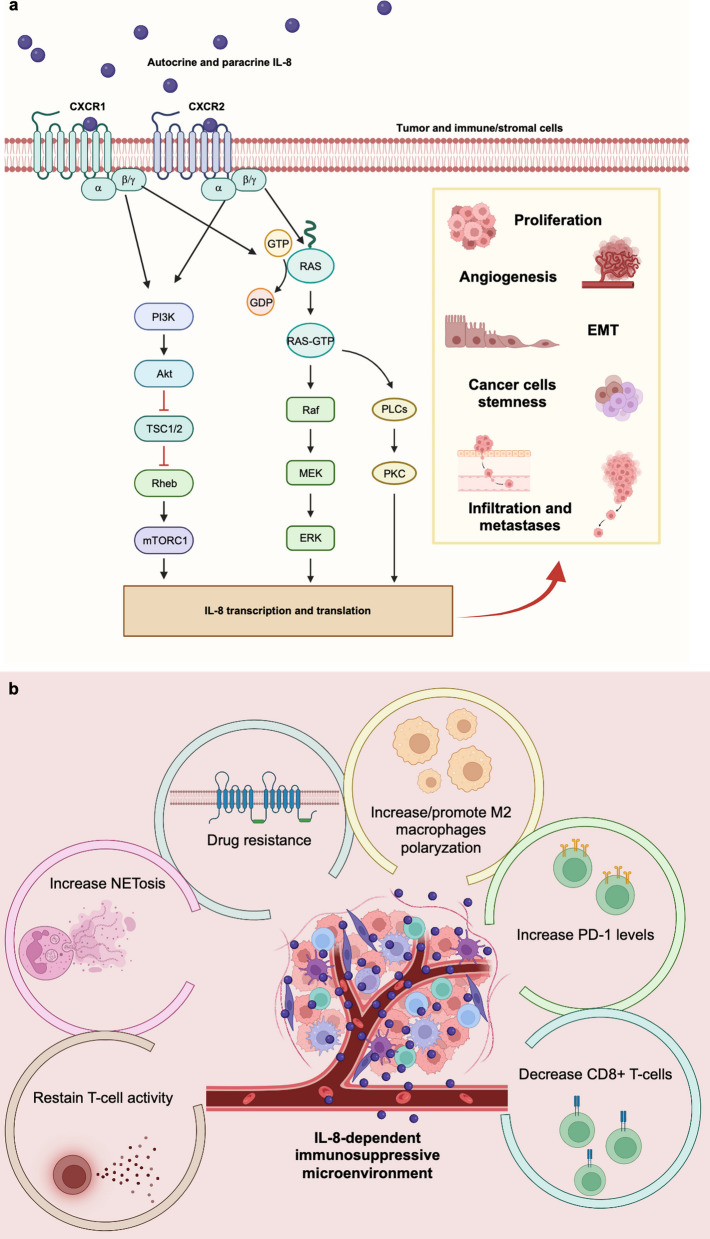
IL-8 protumorigenic and immunosuppressive microenviroment role. Schematic IL-8/CXCR1/2 axis activation in tumor-immune/stromal cells and subsequent induction of protumorigenic processes (**a**). Representative diagram of the main IL-8-mediated features in shaping TiME (**b**)

IL-8 is an ELR + (Glu-Leu-Arg) chemokine; mutations in the ELR residues reduce IL-8 affinity for its receptors (CXCR1 and CXCR2) [30, 31]. Moreover, IL-8 monomers and dimers bind the CXCR1 and CXCR2 receptors with different specificity [32]. Nasser et al*.* showed that the monomer activates both CXCR1 and CXCR2, whereas the dimer selectively activates CXCR2 [32]. Located at 2q34-q35, the *CXCR1* and *CXCR2* genes derive from the duplication of a common ancestral gene and produce two proteins with a 77% of similarity in aminoacidic sequences [33]. The disulfide bonds between cysteine residues in the transmembrane domain 4 and extra cellular loop 2 of CXCR1/2 N-terminus are critical for binding and specificity [34, 35]. According to the N-terminal domains, CXCR1 binds CXCL6 and IL-8, whereas CXCR2 binds CXCL1, CXCL2, CXCL3, CXCL5, CXCL6, CXCL7 and IL-8 [36]. The C-terminus of CXCR1/2 is important for signal transduction and regulates receptor phosphorylation, internalization and G-protein coupling. Details of interactions between IL-8 and CXCR1 and CXCR2 are modeled in [37] and [38], respectively. Phosphorylation and internalization are two critical modulators of CXCR1/2-dependent signaling, due to receptor recycling back to the membrane [10]. CXCR1 and CXCR2 are G-protein-coupled receptors, able to activate intracellular specific molecular cascades. The main molecular downstream axes are the PI3K and MAPK pathways. PI3K is direct target of the heterotrimeric Gαi and βγ subunits and leads to the activation of Akt and mTOR; this in turn promotes cell proliferation, migration and invasion (Fig. 2) [39]. Otherwise, Gαβγ activates the MAPK cascade; downstream transcription factors encode for genes promoting cell proliferation, survival and inflammation [40]. Both the PI3K and MAPK signaling are also involved in the transcription and translation of IL-8, establishing a feedback loop [23, 41]. Another canonical pathway activated by IL-8 is the JAK2/STAT3/Snail signaling pathway, mainly involved in epithelial-to-mesenchymal transition (EMT) [42].

### Physiological functions in health

IL-8 is a central chemokine in healthy physiology and coordinates several key aspects, such as neutrophil behavior, local tissue repair, and vascular responses beyond overt inflammation [43]. In resting tissues, IL-8 transcription is actively repressed (see above); stimuli (such as TNF-α and IL‑1β) lift this repression and induce IL‑8 in stromal cells (fibroblasts, endothelial cells) and leukocytes [44–46]. This controlled, transient production allows IL-8 to fine‑tune local processes like proliferation, survival, adhesion, and repair rather than driving chronic inflammation in healthy organisms [43].

In addition, IL‑8 is one of the most potent neutrophil‑attracting chemokines and directs their migration from blood to tissues by binding the CXCR1 and CXCR2 receptors on neutrophils [47, 48]. It orchestrates every major step of neutrophil extravasation: shedding of selectins (e.g., L‑selectin) and activation of β2 integrins on neutrophils, firm adhesion to endothelium, transendothelial migration, and chemotaxis within tissues toward higher IL‑8 gradients [49–51]. IL‑8 also impacts multiple early neutrophil responses, including membrane depolarization, activation of specific ion transporters, cytoskeletal rearrangement, and degranulation, optimizing rapid microbicidal activity at low systemic cost [6]. Through the IL‑8/CXCR1 axis, it triggers respiratory burst and α‑defensin release, enhancing oxygen‑dependent killing and oxygen‑independent antimicrobial mechanisms in a targeted fashion at sites where it is produced [43, 52]. Finally, IL-8/CXCR2 signaling participates in neutrophil egress from bone marrow, counterbalancing CXCL12/CXCR4‑mediated retention and contributing to the physiological flux of mature neutrophils into the circulation [53]. CXCR2‑based signaling promotes neutrophil “aging” and tissue recruitment, whereas CXCR4 guides aged neutrophils back to bone marrow, so IL‑8 gradients help shape neutrophil subset distribution and homeostatic turnover [53].

Besides neutrophils, IL‑8 acts as a chemoattractant for monocytes, macrophages, mast cells, and to some extent lymphocytes, helping to build a layered leukocyte infiltrate in normal responses to minor tissue damage or infection [47, 54–56]. Bacterial N‑formyl peptides (e.g., N-formyl-methionyl-leucyl-phenylalanine) can override IL‑8‑driven chemotaxis, allowing neutrophils to prioritize direct microbial signals over host‑derived gradients, which refines navigation in vivo and prevents inappropriate tissue infiltration in healthy hosts [57].

In addition to regulating neutrophil functions, IL-8 also plays important roles in tissue repair and remodeling. Endothelial and stromal cell-derived IL‑8 supports wound repair by promoting keratinocyte migration and re‑epithelialization and by recruiting monocytes and neutrophils that clear debris and orchestrate healing [58]. IL‑8 drives angiogenesis by stimulating endothelial proliferation, capillary tube formation, and expression of matrix metalloproteinases (MMP)‑2 and MMP‑9, thereby enabling controlled extracellular matrix remodeling during physiological neovascularization (e.g., wound healing, menstrual cycle) [45].

As highlighted above, stringent transcriptional and post‑transcriptional IL-8 control maintains low baseline levels, preventing unwarranted neutrophil activation and tissue injury under physiological conditions. Conversely, disruption of IL‑8-mediated neutrophil migration is associated with impaired apoptosis and abnormal neutrophil extracellular traps (NET) formation (suicidal NETosis) in pathological conditions (e.g., systemic infections and severe sepsis), underscoring how its normal function is integral to balanced innate immunity [59].

## The pathogenic role of IL-8 in non-malignant diseases

Given its physiological role in the maintenance of neutrophil homeostasis, IL-8 is a key driver of neutrophil‑dominated inflammation across many non‑malignant diseases, linking local tissue injury or infection to excessive or chronic neutrophil recruitment and damage. This is well exemplified by its involvement in chronic airway diseases, such as chronic obstructive pulmonary disease and asthma, and autoimmune and chronic inflammatory diseases, such as rheumatoid arthritis and psoriatic synovitis [60–64]. Below, we briefly discuss the pathophysiological role of IL-8 in sepsis and pathological inflammatory states causing specific tissue damage, such as neuroinflammation, inflammatory bowel diseases (IBD), and cardiovascular and liver diseases.

### Inflammatory bowel disease

IBD are multifactorial, chronic, relapsing inflammatory disorders of the gastrointestinal (GI) tract encompassing two distinct clinical manifestations: Crohn’s disease (CD) and ulcerative colitis (UC). Pathogenesis is driven by a complex interplay between microbial dysbiosis, disruption of the mucus layer, impaired epithelial tight junctions, and increased intestinal permeability. These processes are strongly influenced by genetic susceptibility and environmental factors [65, 66]. The characterization of biopsies of IBD patients revealed that mRNA of IL-8 and IL-17A was higher in IBD patients as compared to healthy subjects [67]. This evidence on IL-8 was confirmed for protein expression, measured in tissue homogenates [68]. The IL-8 analysis in plasma samples confirmed that protein levels are high in active UC and CD patients [69]. A meta-analysis also identified the *IL-8* rs4073 as SNP significantly associated to IBD predisposition [70]. The -91 G > T variant (rs188378669) is more frequent in IBD patients and correlates with higher IL-8 serum concentration, as compared to the *IL-8* −-251 A > T (rs4073) [71]. Recently, it has been shown that the *IL-8* polymorphisms rs103284 and rs105432 are higher in CD patients [72].

The transepithelial migration and infiltration of neutrophils represent a hallmark of IBD, reflecting active mucosal inflammation due to immune system over-reaction to bacteria and tissue damage [73–75]. The chemoattractant IL-8 guides neutrophil migration to sites of epithelial damage in IBD [76, 77]. Microbial products and cytokines, including IL-8, inhibits neutrophils’ apoptosis, increasing their lifespan and blocking the resolution of inflammation [78, 79]. In IBD, neutrophils’ roles are associated with the release of granule-associated mediators, reactive oxygen species (ROS) and NET. NET is a network in which granule proteins, proteolytic enzymes, antibacterial peptides, histones, and other network structures are embedded. In this way, NET disrupts intestinal barrier integrity, promoting the IBD-associated pathological processes [73, 80, 81]. Through CXCR1, IL-8 is also involved in the evolution from IBD to cancer (see below) [82].

### Infectious diseases and sepsis

Sepsis represents a life‑threatening medical emergency, in which a dysregulated host response to infection causes acute organ dysfunction and tissue damage, rather than just controlling the pathogen. It is associated with high mortality rate (~50%) in the elderly, as compared to younger patients (~25%) [83]. Since 1992, Hack et al*.* have described IL-8 alterations in sepsis: high IL-8 plasma levels were observed in septic patients, as compared to healthy donors, regardless of the type of underlying infection (gram positive, gram negative, or both species) [84]. Its concentration correlates with lactate, hypotension, thrombocytopenia, and inflammatory mediators, leading the authors to propose a direct role for IL‑8 in sepsis pathophysiology rather than it being a bystander marker. Ever since, IL-8 has been recognized as a key mediator of sepsis pathophysiology, both as an effector of injury and as a biomarker of disease severity. It is markedly elevated in most patients with sepsis, reflecting strong activation of innate immunity. Through its potent neutrophil‑activating and chemotactic effects, IL‑8 amplifies neutrophil recruitment and degranulation, contributing to endothelial damage, microvascular dysfunction, and organ injury that characterize severe sepsis and septic shock.

Clinically, IL‑8 has been studied as a diagnostic and prognostic biomarker. In adults, high serum IL‑8 levels are associated with the presence and severity of sepsis, and with multiorgan failure and death, resulting more useful for risk stratification and monitoring than for definitive sepsis diagnosis alone. This is true also for septic burn lesions, as compared to non-septic ones, in which IL-8 serum levels are significantly increased from day 5 to day 14 following thermal injury and correlate with significantly greater incidence of sepsis and multiorgan dysfunction [85, 86]. In neonatal/pediatric sepsis, IL‑8 has moderate diagnostic accuracy, with pooled sensitivity and specificity of 0.78 and 0.84 (AUC ≈ 0.89), respectively, with better performance when combined with IL‑6 or other markers [87]. IL-8 levels less than or equal to 220 pg/ml within one day of hospital acceptance also correlate with better survival rates in pediatric patients affected by septic shock [88]. In the elderly population, IL-8 serum levels display a strong prognostic role for mortality, with higher levels in non-survived than survived groups [89]. Not only IL-8 plays a role only in sepsis initiation and progression, but it could also correlate incomplete recovery and chronicization. An example is represented by sepsis-associated encephalopathy, in which the levels of IL-8 in cerebrospinal fluid (CSF) display high diagnostic sensitivity [90].

### Other systemic conditions

#### Neuroinflammation

IL-8 has significant effects within the central nervous system (CNS), particularly during neuroinflammation. IL-8 mainly acts as a strong chemoattractant for neutrophils and is produced by multiple cell types, among which microglia, the brain's resident immune cells, is the primary source [18, 91]. IL-8 receptors are found on neurons, glial cells, and infiltrating immune cells, coordinating complex neuroimmune responses [92–94]. IL-8 production in the CNS is tightly controlled by pro-inflammatory signals, such as lipopolysaccharide (LPS), IL-1β, and TNF-α. Anti-inflammatory cytokines such as IL-4, IL-10, and TGF-β1 can significantly reduce its expression [95, 96]. This regulatory balance is crucial for maintaining appropriate immune responses and preventing excessive neuroinflammation. Under physiological conditions, IL-8 is present at low levels in the CNS, supporting immune monitoring and tissue maintenance. Nevertheless, during states of neuroinflammation, IL-8 production increases significantly, playing a role in various pathological processes, including disruption of the blood-brain barrier (BBB), oxidative stress, and neurotoxicity [97–99]. At the mechanistic level, IL-8 increases BBB permeability by disrupting tight junction proteins, particularly zonula occludens-1, and by forming actin stress fibers in brain endothelial cells via CXCR2 [100]. The expression of CXCR2 on brain endothelium increases with inflammation, with higher levels found in active lesions of multiple sclerosis (MS) compared to healthy tissue [100]. This IL-8/CXCR2 pathway in endothelial cells represents a unique mechanism that directly affects the structure of the neurovascular unit and facilitates further inflammatory cell invasion, independent of immune cell recruitment.

While elevated levels of IL-8 can cause neuronal death by increasing MMP-2 and MMP-9, pro-apoptotic proteins like Bim, and cell cycle proteins such as cyclin D1, other studies point to protective effects [99]. IL-8 has been shown to prevent amyloid-β-induced neuronal death and increase brain-derived neurotrophic factor production in vitro [101]. In cognitively healthy older adults, higher IL-8 CSF levels are associated with better memory performance over time, especially in those with lower phosphorylated τ burden. This suggests a potential protective role when Alzheimer's disease pathology is minimal [102]. Several studies found elevated IL-8 levels in CSF and brain tissue in conditions like Alzheimer's disease, MS, traumatic brain injury, and psychiatric disorders, where its presence correlates with disease severity, demyelination, and ongoing neuronal dysfunction [18, 97, 103–105]. In MS and related demyelinating conditions, IL-8 plays a prominent role in managing immune cell infiltration, demyelination, and tissue damage. IL-8 CSF levels are significantly higher in MS patients, especially during acute relapses, and are associated with disease severity, the extent of spinal cord lesions, and BBB permeability [106, 107].

Pro-inflammatory cytokines, including IL-6, IL-8, and other chemokines, are increased in amniotic fluid, cord blood, CSF, and brain tissue of infants with inflammation-related white matter lesions, mainly periventricular leukomalacia [105, 108].

In autoimmune neuroinflammatory diseases, IL-8 contributes to recruiting immune cells and boosts the inflammatory response, although its role is less prominent than that of IL-1, IL-6, and IL-17 [109].

During ischemic stroke, the IL-8/CXCR2 chemotactic pathway is activated after cerebral artery occlusion, with IL-8 serum levels correlating with infarct volume and functional outcomes [110]. Blood mononuclear cells from stroke patients show increased IL-8 mRNA expression, which correlates positively with IL-8 plasma levels, indicating IL-8's role in recruiting neutrophils to ischemic brain areas [111]. miR-4437 negatively controls CXCR2 expression in peripheral blood leukocytes, and its lower levels in stroke patients lead to increased CXCR2-driven recruitment of granulocytes and NK cells [110].

Additionally, studies on traumatic brain injury indicate that IL-8 is produced by oligodendrocytes near damaged axons, suggesting that IL-8 may also promote myelination, in addition to its role in cell migration [112].

Beyond its pro-inflammatory effects, IL-8 could be a marker for disease activity and treatment response in neuropsychiatric conditions such as major depressive disorder [18]. Nevertheless, its clinical use as a marker is still limited due to variations in methods and other influencing factors.

#### Cardiovascular diseases

IL-8 has emerged as a central inflammatory mediator linking innate immunity to cardiovascular disease, functioning both as a biomarker of vascular inflammation and as an active driver of atherosclerosis progression, plaque instability, myocardial injury and adverse remodeling. IL-8 is produced by several cardiovascular-relevant cell types, including endothelial cells, monocytes/macrophages, vascular smooth muscle cells (SMC), and activated platelets, particularly in response to oxidative stress, lipid accumulation, and pro-inflammatory cytokine stimulation. Through binding to CXCR1 and CXCR2, IL-8 promotes neutrophil chemotaxis and activation, amplifies endothelial dysfunction, and reinforces inflammatory circuits that sustain atherogenesis and vascular injury [113]. In the context of chronic atherosclerosis, IL-8 contributes to leukocyte recruitment and endothelial permeability, facilitating inflammatory cell infiltration into the arterial wall and fostering plaque development. Clinical data support this mechanistic framework, as circulating IL-8 levels have been associated with hyperlipidemia and coronary heart disease even among patients receiving statins, suggesting that IL-8 may capture residual inflammatory risk beyond lipid lowering [113]. Importantly, IL-8 signaling appears particularly relevant in advanced lesions, where persistent chemokine-driven inflammation may contribute to plaque vulnerability and destabilization. In stable coronary artery disease, IL-8 has been shown to independently predict long-term cardiovascular outcomes, consistent with a role in sustaining chronic plaque inflammation and predisposing to acute events [114]. The transition from stable disease to acute coronary syndromes (ACS) is tightly linked to thrombo-inflammation, where IL-8 may act at the intersection of neutrophil activation, platelet function, and endothelial dysfunction. Activated neutrophils release proteases and ROS that can weaken fibrous caps, while platelet-leukocyte interactions further amplify inflammatory and pro-thrombotic responses. Mechanistic evidence indicates that platelet-associated IL-8 is increased after myocardial infarction and correlates with impaired nitric oxide-mediated endothelial responses, supporting the concept that platelet-derived IL-8 contributes to post-infarction vascular dysfunction and pro-thrombotic remodeling [115]. In line with these biological effects, elevated IL-8 levels have been repeatedly associated with adverse prognosis in ACS populations: IL-8 predicts mortality following acute ischemic events and identifies patients with heightened innate immune activation and aggressive inflammatory phenotypes [116].

Beyond the vasculature, IL-8 pathways may also influence myocardial remodeling after ischemic injury, a process characterized by an early neutrophil-dominated inflammatory phase followed by reparative immune responses. Dysregulated or prolonged chemokine signaling can promote maladaptive remodeling, fibrosis, and eventual heart failure. Experimental work suggests that CXCR1/2 may modulate these dynamics: endothelial cells overexpressing IL-8 receptors reduce cardiac remodeling and improve function following myocardial infarction, underscoring the functional relevance of CXCR1/2 signaling in post-injury adaptation [117]. In chronic heart failure, IL-8 has been implicated as a marker of persistent systemic inflammation and a predictor of clinical deterioration. Contemporary cohorts have shown IL-8 to be independently associated with mortality, suggesting that sustained chemokine activation reflects high-risk inflammatory states contributing to disease progression [118]. IL-8 also intersects with broader cardiovascular comorbidities beyond coronary disease and systolic dysfunction. In ischemic stroke, IL-8 is included among key inflammatory biomarkers implicated in endothelial activation and leukocyte recruitment, reinforcing shared mechanisms across cerebrovascular and coronary syndromes [119]. Furthermore, IL-8 signaling may contribute to myocardial and microvascular dysfunction in preserved ejection fraction phenotypes: circulating IL-8 has been linked to invasive evidence of diastolic dysfunction, suggesting chemokine-driven inflammation as a potential contributor to myocardial stiffening and impaired relaxation [120].

Collectively, these findings position IL-8 at the crossroads of endothelial dysfunction, leukocyte recruitment, platelet-neutrophil crosstalk, and inflammatory remodeling, providing a unifying chemokine axis across atherosclerosis, acute ischemic events, and heart failure progression.

#### Liver diseases

Many studies have established the pathogenetic role of IL-8-mediated pathways in both acute and chronic liver disease, as IL-8 signaling has been shown to be involved in acute liver injury during ischemia-reperfusion stages [121]. Acute alcoholic hepatis is a severe form of hepatic necro-inflammatory process, characterized by hypertransaminasemia, neutrophilia, jaundice, coagulopathy, hepatic encephalopathy and liver failure. In this form of hepatitis, IL-8 serum levels are significantly higher compared to other liver disease etiologies (metabolic and viral). In addition, IL-8 is strictly correlated with histologic disease severity, plasma liver biochemistry (transaminases, bilirubin), circulating neutrophils, development of portal-hypertension related complications and short and mid-long term liver-related mortality [122–124]. In this context, IL-8 is a causative agent for hepatic infiltration by immune cells (neutrophils, macrophages, NK) that cause parenchymal dysfunction and could be a pharmacological target for future studies [124].

The pathogenetic role of IL-8 has been also evaluated in chronic liver diseases. In a study involving 200 patients, IL-8 plasma levels and intra-hepatic expression of *CXCR1/2* genes are progressively higher with worsening severity of liver cirrhosis and strongly correlate with liver function, pro-inflammatory cytokine levels and liver fibrosis [125]. In cholestatic diseases and cholestatic damage associated to various liver disorders, the pathogenetic role of IL-8 was confirmed by detecting a markedly high expression in reactive bile ductules [126]. Metabolic-associated steatotic liver disease represents a model in which genetic, environmental and metabolic factors determine chronic inflammation and, ultimately, liver fibrosis. In mice, IL-8 induces neutrophil-mediated oxidative burst, collagen deposition and hepatic stellate cells activation [127]. In humans, IL-8 production, together with osteopontin and monocyte chemoattractant protein 1, strongly predicts advanced hepatic fibrosis [128].

## The dichotomy of IL-8 in oncogenesis and cancer progression

The malignant growth of cancer cells induces and is, in turn, modulated by a profound reorganization of the tumor immune microenvironment (TiME). In this complex and multi-step process, among other cytokines/chemokines, IL-8 plays a central role as an autocrine and paracrine soluble factor (Fig. 2).

### Protumorigenic mechanisms of the IL-8/CXCR1/2 Axis

IL-8 has a multifaceted role in cancer progression. By binding to its CXCR1/2 receptors, expressed on the surface of tumor and stromal/immune cells, it activates both autocrine and paracrine signals. PI3K and MAPK pathway activation downstream of receptor engagement (and sometimes EGFR trans‑activation) promote the activity of transcription factors such as NF-κB, HIF-1 or AP-1, thereby triggering cell proliferation, invasion, angiogenesis and metastases (Fig. 2) [129].

In addition to its role in the regulation of survival and functional “fitness” of neutrophils and other myeloid cells, as well as to its ability to support hematopoietic and myeloid‑derived suppressor cell (MDSC) populations from bone marrow, the IL‑8/CXCR1/2 axis may act as an autocrine growth factor in many cancers (e.g., melanoma, breast, lung, CRC), increasing DNA synthesis, clonogenicity, and expansion of the malignant clone [130–132]. Neutralizing or silencing IL‑8 in melanoma and other tumor models inhibits proliferation, while adding recombinant IL‑8 rescues growth can helps dormant cells re‑enter the cell cycle and regrow at metastatic sites [7, 10, 133–135]. Multiple lines of evidence also establish IL-8 as a modulator of stem cells features, for example in CRC models, where it controls the expression of stem cell markers (Oct-4, CD44, EpCAM, Lgr5) [136]. On the other hand, the expression of stemness markers may be correlated with IL-8 production: for example, Oct-4 overexpressing CRC cells stimulate the production of IL-8, thereby promoting tumorigenesis [137]. Interestingly, in esophageal squamous cell carcinoma (ESCC) the expression of NEDD9 in cancer stem cells drives ERK-dependent IL-8 release, leading to the recruitment of granulocyte-like MDSC; MDSC, in turn, promoted ESCC stemness via the Notch pathway [138].

By activating Rho‑GTPases and downstream cytoskeletal remodeling (e.g., actin polymerization and lamellipodia formation), IL‑8/CXCR1/2 signaling increases cancer cell motility and invasion into surrounding stroma [139, 140]. In non‑metastatic melanoma or head‑and‑neck cancer, overexpression of IL‑8 increases migration and invasion through basement‑membrane substitutes in vitro and metastatic potential in vivo [141, 142]. In melanoma models, UV-B exposure enhances melanoma cell motility via induction of autocrine IL-8 secretion [143]. IL-8 expression is also able to enhance CRC progression, by affecting cell migration and invasion, through the upregulation of the integrin αvβ6 [144]. Chen et al*.* showed that in pancreatic cancer IL-8 expression correlates with MMP-1, as observed in the public datasets TGCA and GEO (GSE28735), and that hsa-miR-623 reduces IL-8 and MMP-1 expression and metastasis [145]. Moreover, in pancreatic cancer cell lines, IL-8 siRNA- nanoplexes reduce cell invasion and migration, and induce apoptosis [146]. In gastric cancer (GC), IL-8 levels increase under the selective pressure of chemotherapy and IL-8 overexpression marks the onset of chemoresistance. In that context, IL-8 silencing by siRNA inhibits proliferation and migratory ability of tumor cells [147]. IL‑8 signaling is also tightly connected to EMT, one of the most characterized mechanisms regulating invasive and metastatic ability, linked to the activation of specific transcription factors, such as Slug, Snail, Twist and β-catenin [148–150]. Tumor cells undergoing EMT upregulate IL‑8 and its receptors, and IL‑8 in turn is required to maintain their mesenchymal, invasive phenotype [151]. IL‑8 can induce EMT programs in neighboring epithelial tumor cells via pathways such as PI3K/Akt, TAK1/NF‑κB, and Wnt/β‑catenin, increasing expression of transcription factors like ZEB1 and thereby promoting collective migration and deeper tissue invasion [139]. In both renal cell carcinoma (RCC) and GC cells, IL-8 induces EMT by enhancing N-cadherin and reducing E-cadherin expression through activation of the PI3K pathway, inhibiting apoptosis, and inducing MMP expression [139, 152].

Within the tumor stroma, IL-8 increases the VEGF mRNA transcription, as well as regulates VEGF-independent angiogenesis [153]. This mechanism is related to the induction of proliferation and survival of endothelial cells and release of MMP-9 and MMP-2 [45, 134]. In mouse models, IL-8 enhances CD31^+^ peritumoral vasculature in a CXCR2-dpendent manner [154]. Moreover, TRAIL^+^ non-small cell lung cancer (NSCLC) cells express high levels of IL-8 and mRNA expression of both TRAIL and IL-8 positively correlate with the angiogenesis marker CD31 [155]. This evidence clearly establishes IL-8 overexpression as a resistance tool upon VEGF inhibitors treated patients, even in VEGF-driven cancers, such as RCC. Indeed, different clinical trials reported the associations between high baseline circulating IL-8 levels and shorter overall survival (OS) during VEGF inhibitors schedule [156]. By acting on CXCR1 and CXCR2 receptors on neutrophils, low IL‑8 gradients guide chemotaxis into inflamed or tumor tissues, while much higher, receptor‑saturating IL‑8 levels trigger NETosis, leading to extracellular DNA-protein webs rich in granule enzymes and proteases that remodel matrix and activate endothelium [157]. NET themselves have pro‑angiogenic properties. They carry myeloperoxidase (MPO), elastase and MMP‑9, can increase endothelial adhesion molecule expression, and contribute to endothelial activation and sprouting, thereby complementing the direct pro‑angiogenic effects of IL‑8 on endothelial migration and tube formation [158, 159]. Importantly, NETosis is completely abolished in the presence of the IL-8 neutralizing antibodies [160, 161].

### Shaping an immunosuppressive tumor microenvironment

IL-8 can be released by all the cell populations characterizing the TiME (Fig. 2). In hepatocellular carcinoma (HCC) patients, high levels of IL-8 and low levels of catalase significantly correlate with increased overall immune cells infiltration, but reduced levels of naïve CD8^+^ T cells and worse prognosis, as compared to IL-8 low/catalase-high cases [162]. Upon IFN-α stimulation HCC cell lines upregulate IL-8 production, which restrains T cell activity, raising the hypothesis that IL-8 targeting may potentiate immunotherapy [163]. In metastatic urothelial carcinoma and RCC, high levels of IL-8 in plasma, peripheral blood mononuclear cells, and tumor cells correlate with reduced clinical efficacy of the anti-PD-L1 atezolizumab, counteracting even the "inflamed" tumor condition characterized by a T_eff_ transcriptomic signature associated with better response to immunotherapy [164]. Overall, one the major obstacles to immunotherapy efficacy is the depletion of CD8^+^ T cells. In melanoma, IL-8 expression correlates positively with NET and negatively with CD8^+^ T cells density [165]. Xu et al*.* demonstrated that exosomes derived from prostate cancer cells transport IL-8, which enhances T cell starvation and exhaustion by altering fatty acid oxidation and glucose metabolism [166]. Li et al*.* showed that high level of IL-8 serum correlates with poor prognosis in GC: IL-8 released by cancer-associated fibroblasts (CAF) enhances the expression of PD-1 in CD8^+^ T cells populations and immunosuppression [167]. Zhai et al*.* showed that IL-8 produced by CAF activates NF-κB signaling in GC cells and upregulates the ATP-binding cassette subfamily B member 1 involved in drug resistance [168, 169]. Lin et al*.* demonstrated that IL-8 is expressed by PD-L1^+^ macrophages, one of the main populations infiltrating IL-8 high GC, resulting in reduced CD8^+^ T cells and an immunosuppressive microenvironment. Consistently, the CXCR1/2 inhibitor reparixin significantly reduces PD-L1^+^ macrophages and increases apoptotic signals in epithelial cells [170]. Sun et al*.* showed that IL-8 produced by mesenchymal stem cells is responsible for PD-L1 expression by GC cells, which protects them against the cytotoxic effects of CD8^+^ T cells [171]. Shao et al*.* showed that M2 macrophage polarization in CRC is under the control of the IL-8/STAT3 axis; moreover, IL-8 induces PD-L1^+^ M2 macrophages to infiltrate tumors, thereby blocking cytotoxic PD-1^+^/CD8^+^ T cells [172]. IL-8 produced by pancreatic tumor cells increases tumor infiltration by CXCR2^+^/CD68^+^ M2 tumor-associated macrophages (TAM) in murine models, thereby causing an immunosuppressing environment. High percentage of CXCR2^+^/CD68^+^ macrophages were similarly detected in the peripheral blood of pancreatic cancer patients. IFN-γ suppresses IL-8 and other tumor-derived soluble factors. Hence, the use of IFN-γ increases the effect of PD-1 blockade through disruption of the IL-8/CXCR2 axis and consequent reduced infiltration by CXCR2^+^/CD68^+^ macrophages [173]. In lung cancer, CAF release IL-8, which recruits TAM or promote M2 macrophages polarization; this effect is rescued by the use of the CXCR2 inhibitor SB225002 that increases the effectiveness of anti-PD-1 and anti-PD-L1 treatment in mouse models [174]. In addition, in lung cancer patients with high infiltrating macrophages, IL-8 mRNA is upregulated in cancer cells interacting with macrophages [175]. Zhong et al*.* recently demonstrated that the TAM infiltration enhances pancreatic cancer progression through IL-8 release. Through activation of the STAT3 pathway, TAM produce IL-8, which in turn induces glycolysis; this effect, observed both in vitro and in vivo, is specifically related to IL-8, as it can be reversed by the CXCR1/2 inhibitor reparixin [176]. Schimek et al*.* also demonstrated that apoptotic CRC cells attract neutrophils in the TiME via IL-8 release and that infiltrating neutrophils induce an M2-like macrophage phenotype [177]. Song et al*.* showed that IL-8, released by M2 macrophages under hypoxic conditions, increases cell proliferation, EMT, migration, invasion, and PD-L1 expression in esophageal cancer (ESCA) cells [178]. Olivera et al*.* showed that peripheral blood T cells induce IL-8 production from CRC cells in vitro, in an IL-1β and TNF-α dependent manner. Blocking the activity of these cytokines (using anakinra, infliximab, and etarnecept) interrupts such pathogenic cytokine loop also in paired plasma samples of patients undergoing TNF-α blockade with infliximab in a clinical trial [161]. It is also important to consider that many of the mechanisms described are also induced by cellular senescence which induces IL-8. Indeed, IL-8 is a component of the senescence-associated secretory phenotype (SASP). Although SASP has been historically regarded as tumor-suppressive by enforcing cell-cycle arrest in senescent cells, the secretion of multiple cyto-/chemokines -including IL-8- can promote a pro-tumorigenic microenvironment [179]. This activity may explain the association between IL-8 and an immunosuppressive TiME that contributes to ICI resistance [180]. For example, IL-8 secreted by senescent mesenchymal stem cells has been reported to upregulate PD-L1 in GC, impairing CD8^+^ T cell cytotoxicity [171]. IL-8-mediated upregulation of HLA-E can further inhibit CD8^+^ T and NK cell function [181]. Additionally, as a SASP component, IL-8 recruits Th2 cells and promotes M2 macrophage polarization [182].

While the bulk of evidence supports a pro-tumorigenic role for IL-8, whether produced by cancer or immune cells, a few discordant observations point to the possibility that local production of IL-8 in CRC may actually contribute to the control of tumor growth, possibly through immune activation. Li et al. have indeed identified the *IL-8* gene in a high-ImmuneScore population that contributed to better survival CRC. Further studies revealed IL-8 association with the DC activation markers CD80, CD83, and CD86, suggesting an IL-8-mediated antitumor immune response, related to its ability in DC recruitment and activation in CRC tissues. In vivo, CXCR2 blockade promotes CRC progression mediated by reduced activated DC infiltration and decreased expression of IFN-γ or granzyme B by CD8^+^ T cells. [183]. Consistently, IL-8 expression in the tumor infiltrate correlated with earlier disease stage (p < 0.001) and improved relapse free survival (RFS) across a CRC cohort (p < 0.001) and was independently associated with enhanced RFS in multivariate Cox regression analysis [184]. Recently, we characterized 168 CRC specimens and showed a significant correlation between lack of tumor-infiltrating IL-8^+^ mononuclear cells and PTEN-loss in cancer cells, a molecular trait associated with resistance to EGFR‑targeted treatments and reduced antitumor immune response in CRC [185, 186]. Available evidence establishes a link between IL-8, mismatch repair (MMR) proficiency (pMMR) or deficiency (dMMR) status, and expression of the PD-L1/PD-1 immune checkpoint [171, 187, 188].

IL-8 ability to modify the immune infiltrate of cancer tissues hence exerts a profound influence on response to immunotherapy. Indeed, from a mechanistic translational point of view, this pleiotropic IL-8 expression in TiME acts as a consequent obstacle to favorable outcomes under immunotherapy treatment. The progression of cancer disease promotes IL-8 increase by cancer, stromal and immune cells. Overall, ICI-based immunotherapy mainly acts by blocking the PD-1/PD-L1 binding, the inhibitory signal of cytotoxic lymphocytes. In the presence of IL-8, CD8^+^ T cells are depleted, mainly by the recruitment of MDSC acting as promoter of immune-mediated tumor evasion and inhibitor of cytotoxicity of immune cells [189]. This mechanism is sustained by the production of IL-8 by infiltrating MDSC, impairing the machinery of antigen presentation [164]. The remain CD8^+^ T lymphocytes reduce the expression of PD-1. IL-8 also recruits M2 macrophages expressing PD-L1, which are able to switch off lymphocytes’ activity, through PD-1 binding. Moreover, IL-8-induced NET form a physical trap around tumor cells. These IL-8-mediated mechanisms prevent the destroy of cancer cells by cytotoxic lymphocytes, hence resulting in ICI resistance. Together with the trigger of cancer cell proliferation, angiogenesis and metastasis, IL-8 sustains the survival of cancer cells and immunotherapy resistance.

These IL-8-mediated mechanisms highlight the clinical need to identify IL-8 blood‑based signatures, by which clinicians can actually define subpopulations of cancer patients who benefit from ICI treatment.

### Interplay of IL-8 with gut microbiota

Although it is still incompletely clarified, the interplay between IL-8 and gut microbiome may contributes to cancer progression and tumorigenesis. Gut dysbiosis and chronic inflammation converge to create a pro-tumorigenic microenvironment [190]. Pathogenic bacteria within the gut ecosystem initiate this cascade through multiple mechanisms: direct bacterial invasion of malignant cells, disruption of the integrity of the intestinal epithelial barrier, and activation of pattern recognition receptor signaling by bacterial components [191, 192]. Specifically, microbial products such as LPS, flagellin, and peptidoglycan fragments activate TLR4, TLR5, and NOD-like receptors on intestinal epithelial cells and myeloid populations, leading to sustained activation of NF-κB and MAPK pathways and transcriptional upregulation of *IL-8* [193]. Notably, several bacteria directly prompt IL-8 production. *Fusobacterium nucleatum* utilizes its surface adhesin Fap2 to invade CRC cells and CAF, selectively inducing IL-8 and CXCL1 secretion that drives robust cancer cell migration and metastatic potential [194, 195]. Similarly, *Streptococcus bovis* triggers an inflammation-driven tumorigenic sequence involving the coordinated interplay of IL-1, IL-8, and cyclooxygenase-2 [196]. In pancreatic ductal adenocarcinoma (PDAC), the intratumoral microbiome is strongly influenced by microbial migration from the gut and the oral cavity. Intratumoral dominant *Proteobacteria* directly induce pro-tumorigenic IL-8 and IL-6 production, from both neoplastic and pancreatic epithelial cells, thereby modulating both epithelial and stromal compartments to serve as mediators of tumorigenesis [197, 198].

The immunomodulatory potential of gut microbiota also relies on the production of short-chain fatty acids (SCFA; e.g., butyrate, propionate, acetate). SCFA are pivotal in maintaining the anti- and pro-inflammatory balance downregulating the secretion of specific inflammatory cytokines [199]. Consequently, depletion of SCFA-producing commensals (e.g., *Faecalibacterium prausnitzii*) removes epigenetic anti-inflammatory constraints on *IL-8* transcription, further reinforcing IL-8-dependent circuits [200, 201]. IL-8 increase, in turn, perpetuates a self-reinforcing cycle wherein this chemokine facilitates immune cell recruitment to the TiME, promotes angiogenesis through endothelial cell activation, enhances cancer cell proliferation and survival via ERK/NF-κB signaling pathways, and augments metastatic dissemination through increased cellular motility [27].

The impact of the microbiome/IL-8 axis may also extend beyond GI malignancies to influence distant tumor sites. In endometrial and ovarian carcinomas, microbial dysbiosis is associated with increased levels of inflammatory cytokines, including IL-6, IL-8, and IL-17, affecting local TiME and tumor growth [202, 203]. In addition to the gut microbiota, the oral microbiota may also influence carcinogenic and immune processes beyond the GI tract. Oral microorganisms can be translocated to the gut and subsequently reach the systemic circulation, where pathogenic species, including periodontal pathobionts, may disrupt intestinal microbial homeostasis and trigger immune responses, ultimately contributing carcinogenesis in the lung [204]. In particular, *Porphyromonas gingivalis* induces the production of pro-inflammatory cytokines such as IL-1, IL-6, TNF-α and IL-8. IL-6 and IL-8 interact with lung epithelial cells via NF-κB-dependent pathways, thereby promoting tumorigenesis [205].

Whether IL-8 signaling may directly influence gut microbiota composition and function is still incompletely elucidated. Most evidence is indirect and derives from interventional studies rather than from mechanistic investigations. For example, dietary mannose has been shown to: reduce *IL-8* transcription by targeting the OGT/hnRNP R/JUN/IL-8 signaling axis; increase the proportion of beneficial bacteria (i.e., *Lactobacillus intestinalis*, *Lactobacillus acidophilus*, and *Ligilactobacillus salivarius*) in the gut microbiota of NSCLC-bearing mice; enhance anti-inflammatory metabolite production in both systemic circulation and fecal compartments [206].

Although robust evidence for a direct bidirectional relationship between IL-8 and the gut microbiota is still limited, the complex network linking microbial dysbiosis, inflammatory cytokines, immune cell infiltration, and TiME composition and function highlight the important role of the microbiome/IL-8 axis in carcinogenesis. This interplaypan-cancer perspectivecould contribute to malignant transformation, tumor progression, and the development of therapeutic resistance across a wide range of cancer types, including those arising in the GI tract.

### A pan-cancer perspective on IL-8

The role of chronic inflammation and the potential prognostic/predictive role of IL-8 expression have been studied in many different solid tumors, particularly in the setting of systemic immunotherapy [207]. Among these, GI, lung, and kidney cancers, as well as melanoma have been extensively studied [185, 208–211]. GI cancers account for approximately 5.3 million new cancer cases and 3.7 cancer-related deaths per year globally, with esophagus, stomach, CRC, pancreas and liver providing the bulk of this burden [212]. Lung cancer accounts for approximately 2.5 million new cancer cases and 1.8 cancer-related deaths per year globally, remaining the leading cause of cancer-related deaths worldwide [213, 214]. Albeit much less frequent, kidney cancer and melanoma combined account for over 700 000 new cases and approximately 240 000 deaths globally per year [215, 216]. Although these figures vary widely by region, health‑system funding, and time period, the approximate proportion of patients worldwide ever receiving immunotherapy is: 5%-10% for GI cancers; 30%-40% for lung cancer, with much higher rates in high‑income settings and very low in low‑income settings; 25%-35% for kidney cancer, with figures likely > 70% among metastatic RCC in well‑resourced systems; 30%-40% for melanoma, again with strong dependence on system resources and increasing usage in advanced/metastatic cases (> 70%-80%).

#### Expression of IL-8 and its receptors (CXCR1 and CXCR2) in solid tumors

In ESCA, both IL-8 and CXCR1/2 expression are higher in tumor tissue than in paired normal esophageal biopsies or adjacent normal epithelium [217, 218]. Moreover, systemic up‑regulation of IL-8 expression linked to the presence of esophageal malignancies is supported by elevated levels of this chemokine in the serum of patients with ESCC and esophagogastric junction adenocarcinoma, as compared to healthy controls [219, 220]. In GC, IL-8 and its receptors (CXCR1 and CXCR2) are upregulated in cancer tissue (in both intestinal and poorly cohesive subtypes), as compared to normal mucosa, supporting cancer cell responsiveness to an IL-8 dependent autocrine loop [221–224]. Moreover, CXCR2 is overexpressed by stromal cells in GC and its expression on bone-marrow derived mesenchymal cells is induced by conditioned medium from diffuse-type GC cell lines [225]. Overexpression of the IL-8/CXCR2 axis has been extensively documented by our and other groups in CRC [185, 226, 227]. Multiple studies show that IL‑8, CXCR1 and CXCR2 are upregulated in pancreatic cancer (in both adenocarcinoma and neuroendocrine tumors) compared with normal pancreas [228, 229]. Deng et al*.* demonstrated that HCC tissue displays high IL-8 protein as compared to non-tumoral counterpart [230]. According to IL-8 protein levels, the metanalysis by Shakiba et al*.* showed that also IL-8 serum is higher in HCC patients as compared to healthy patients or patients affected by chronic hepatitis and liver cirrhosis [231]. Since 2013, preclinical studies in HCC cell lines demonstrated that IL-8 modulates malignant characteristics such as growth and metastases [232]. IL-8 and CXCR2 appear to be overexpressed in a subset of cholangiocarcinoma (CCA; both hilar and intrahepatic primary locations) cases [150, 233, 234]. Tong et al*.* showed an increase of IL-8 levels in cancer tissue, as compared to adjacent normal tissue, in lung adenocarcinoma [235]. Multiple studies indicate upregulation of IL‑8, CXCR1 and CXCR2 in RCC compared with non‑tumor kidney tissue. In particular, intratumoral IL‑8 is significantly higher in clear‑cell RCC (ccRCC) than in adjacent peritumoral/normal tissue and is further enriched in metastatic cases, where it associates with features of EMT [152, 236]. ccRCC harbors subpopulations expressing both high IL‑8 and high CXCR1, exhibiting stem‑like properties [237, 238]. CXCR2 expression is increased in RCC tissue and is further augmented by TNF‑α [239]. IL‑8 is low or absent in benign/early lesions and normal melanocytes, while many primary and especially metastatic melanomas express higher IL‑8 mRNA and protein [132, 240]. While CXCR1 is reported as constitutively expressed on melanoma cells, CXCR2 expression is particularly associated with more aggressive, highly metastatic tumors [241, 242].

#### Prognostic role of IL-8 in cancer

Clinical data have established IL-8 as an easily detectable blood marker predicting the prognosis of cancer patients. More controversial data are reported. In blood and tissue, SNP can be genetically characterized by liquid biopsy using both circulating tumor DNA (ctDNA) and cells (CTC), allowing for non-invasive and longitudinal monitoring during treatment. In tissue, SNP can be analyzed using traditional sequencing techniques such as NGS or PCR. The major obstacle of the SNP clinical use is that some associations with functional consequences have been only hypothesized [21]. On the other hand, tumor tissue can also be characterized at the mRNA level, by RT-qPCR and RNA-seq. In this context, the limitations derive from the loss of the structural architecture of TiME and hence the possibility to define which cell population has actually expressed IL-8. In plasma or serum, IL-8 protein can be quantified using ELISA assays. This analysis of circulating IL-8 in blood renders its detection standardized and minimally invasive, making it an ideal tool for dynamically monitoring treatment response. In tissue, IL-8 protein can be detected by IHC, despite today advanced methods, such as multiplex IHC (mIHC) and spatial transcriptomics could represent more clinically relevant techniques.

As a result, the type of clinical information extracted can be highly controversial, as these represent different aspects of tumor biology. In particular, circulating IL-8 levels may reflect the overall tumor burden and/or systemic inflammation, whereas tissue IL-8 explain TiME interactions [243]. Therefore, it is necessary not only the standardization of methodology and source, but also the information that clinicians and researchers want to extract. Indeed, as blood measurement can be clinically validated prognostic/predictive biomarker, tissue analysis should be investigated to defined IL-8-dependent TiME interactions behind ICI resistance/sensitivity (Table 1).

**Table 1:** Overview of the critical points in standardizing IL-8 detection in cancer

Source of IL-8	IL-8 molecule	Methodology of IL-8 detection	*Pro*	Co*ntro*
Blood	DNA	ctDNA/CTC	Minimally invasive; longitudinal monitoring	Not all SNP are associated with a functional consequence
	Protein	ELISA	High specificity; standardized protocols; minimally invasive; longitudinal monitoring	No multiplexing
		Multiplex electrochemiluminescence	High specificity; simultaneous analysis of multiple markers	Specialized and expensive equipment
Tissue	DNA	NGS/PCR	High specificity	Not all SNP are associated with a functional consequence
	mRNA	RT-qPCR/RNA-seq	Standardized protocols	Loss of spatial architecture; inability to map the source of IL-8 production
	Protein	IHC	Identification of therapeutic targets; maintenance of spatial architecture	Lack of standardization of the primary antibody; subjective interpretation; inability to map the source of IL-8 production
		mIHC/spatial transcriptomics	Mapping the source of IL-8 production; sparing tumor tissue; maintaining spatial architecture	Elevated costs/time for analysis

Despite the lack of standardization, the comparison of different scientific studies aimed at characterizing IL-8 as a prognostic and/or predictive factor has generally shown a concordance between IL-8 measured in blood and tissue in some solid tumors (Table 2).

**Table 2:** Negative prognostic/predictive association value of IL-8 in several solid malignancies

Type of cancer	Negative prognostic value of IL-8			Negative predictive value of IL-8		
	**SNP**	**Circulating**	**Tissue**	**SNP**	**Circulating**	**Tissue**
Esophagus	✓		✓		✓	
Stomach	✓	✓				
Colon-rectum	✓	✓		✓		
Pancreas		✓				
Liver			✓	✓	✓	
Lung		✓			✓	
Kidney		✓		✓	✓	
Melanoma		✓			✓	

IL-8 expression (both at mRNA and protein levels) has been reported as a prognostic biomarker in ESCC [219, 244]. Ogura et al. demonstrated that the positive expression of both IL-8 [measured in tissues by immunoistochemistry (IHC)] and CXCR2 correlates with low survival rates [both OS and RFS] in surgically resected ESCC patients [245, 246]. However, data obtained on IL-8 mRNA levels don’t correlate with overall patients’ outcome. Such discrepancy can be related to the different methods used to measure IL-8 (protein and mRNA, respectively). Elevated circulating IL-8 levels correlate with higher tumor stage and increased risk of lymph node metastases, as well as with poor chemotherapy response, in GC patients [168, 169, 247]. Again, there is not a significant correlation between high *vs* low tissue IL-8 mRNA levels in terms of both OS and disease free survival (DFS) [170]. Whether such discrepancy is due to the source of IL-8 detection (plasma/serum *vs* tissue) or the different methods used to measure IL-8 (protein and mRNA, respectively) remains to be established. Conversely, high levels of tissue IL-8 mRNA correlate with better OS, as compared to patients with low levels, but show no significant correlation with DFS. Two meta-analyses published in 2014–2015 correlated high levels of serum IL-8 with poor prognosis in CRC patients [226, 248]. In an updated meta-analysis recently published by our group, circulating IL-8 appears to support a more solid prognostic value, as compared to tissue-detected IL-8 [185]. While technical issues (source and method of detection) can still explain such discrepancies, subtler biological reasons related to the multifaceted role of IL-8 in dictating tumor-stroma interactions and TiME composition and function may be hypothesized. As discussed above, local IL-8 production in CRC tissues can lead to DC retention and activation, resulting in an IL-8/CXCR2-stimulated cytotoxic anti-tumor response [183]. Consistently, IL-8 expression in the tumor infiltrate correlates with a PTEN-competent status of CRC cells, earlier disease stage and improved RFS [184, 185]. In pancreatic cancer patients, IL-8 serum levels are associated with shorter OS and DFS and poor prognosis [249]. The IL-8 receptor CXCR1 is specifically expressed in PDAC tumor tissue and significantly associated with lower survival rate and lymph node metastases [250]. Consistently, it has been recently demonstrated that IL-8 can be used as biomarker to distinguish metastatic from locally advanced patients, suggesting IL-8 as a tool for predicting disease trajectory and patients' prognosis, with potentially better performance as compared to canonical tumor markers, such as CA19-9 or CEA [251, 252]. At an apparent difference with CRC (see above), the concomitant presence of IL-8^+^ tumor-infiltrating inflammatory cells and high IL-8 circulating levels are associated with worse prognosis in PDAC [253]. However, high expression of IL-8 mRNA in pancreatic cancer tissues do not result in significant OS and DFS differences. Tissue IL-8 expression more consistently represents a negative prognostic factor in HCC: high levels of tissue IL-8 correlate with advanced stage and high tumor infiltration, and with increased risk of death and recurrence [230, 254]. It is well known that tissue IL-8 expression, detected by IHC, also correlates with unfavorable prognosis of CCA. Moreover, this prognostic value is reinforced by the fact that IL-8 correlates with higher levels of MMP-9 and microvessels density which contribute to aggressiveness of CCA [233]. Data confirm that high mRNA IL-8 expression is related to a worse OS, but not DFS, in liver cancer. Taken together, these results highlight consistent analyses between mRNA and protein IL-8 in liver cancer prognosis. Our group recently published two meta-analyses on the prognostic role of IL-8 in lung and kidney cancers, respectively. High IL-8 serum levels correlate with worse OS in lung cancer and with both worse OS and worse PFS in RCC [255, 256]. No prognostic correlation of IL-8 levels and OS/DFS is observed in lung cancer patients. On the contrary, a prognostic value of tissue expression of IL-8 mRNA shows a correlation between high IL-8 levels and worse OS. Few studies have described the prognostic role of IL-8 in melanoma patients. Overall, elevated serum IL-8 correlates with higher tumor burden and disease stage in melanoma specimens [243, 257]. Kucera et al*.* showed that higher IL-8 plasma levels correlate with increased Breslow stage [258]. No significant differences between high *vs* low IL-8 expressing patients in terms of OS and PFS have been observed.

#### Predictive role of IL-8 in cancer

An overview of validated IL-8 detection as a negative predictive tool is reported in Table 2. Given the established role of IL-8 in shaping an immunosuppressive TiME, IL-8 has been investigated as a rational biomarker to predict response to immunotherapy in cancer patients. Clinical application of immunotherapy in GI cancers is still relatively limited, hence few data on IL-8 as a predictive biomarker are available. Gao et al*.* studied predictive biomarkers of immunotherapy in the plasma of 91 ESCA patients, collected before and post treatment, obtaining an angiogenesis-related risk score. The authors demonstrated that IL-8 is one of three angiogenesis-related proteins, and its baseline levels are associated with both shorter OS and PFS [259]. A recent metanalysis by Zou et al*.* identified IL-8 as a prognostic/predictive biomarker for ICI-based therapy in multiple solid tumors. Patients with low IL-8 protein, detected in either tissue or blood, show more pronounced sensitivity to immunotherapy (i.e., improved OS, PFS, and overall response rate); consistently, IL-8 serum levels appear to decrease during immunotherapy treatment in responder patients [211, 260]. Zhang et al*.* observed that serum IL-8 represents a negative predictive biomarker for immunotherapy in 80 advanced HCC patients. More specifically, early IL-8 increase after the second dose of immunotherapy could predict worse patients OS and PFS, demonstrating the importance of monitoring serum IL-8 to predict therapy efficacy [261]. These data have been recently confirmed in a cohort of 200 HCC patients [262]. Little is known about the role of IL-8 as a modulator of response to immunotherapy in the setting of MSI/dMMR, characterized exquisite sensitivity to ICI. Interestingly, our group has recently reported two cases of advanced head-and-neck squamous cell carcinoma (HNSCC), characterized by a similarly high tumor mutational burden, sustained by MSI in one case that displayed a long-lasting complete response to the anti-PD1 nivolumab, but not in the second case, which demonstrated primary refractoriness to nivolumab. The ICI-responsive, MSI case displayed prominent infiltration of the tumor by immune cells, including IL-8^+^ macrophages, while the ICI-resistant case showed an immune-deserted TiME [263, 264]. Similarly, we recently observed a case affected by the Muir-Torre variant of Lynch's syndrome (characterized by germline mutations in MMR genes), who developed a breakthrough GC, while in complete response to the anti-PD1 pembrolizumab for metastatic duodenal adenocarcinoma [265]. In this interesting case, IL-8^+^ TAM were detected in the ICI-responsive duodenal adenocarcinoma and lost in the immune-refractory gastric lesion (Zen V et al*.*, manuscript in preparation). More consistent data have been obtained regarding the role of IL-8 in predicting response to immunotherapy in lung and kidney cancer and melanoma. A recent study by Akamatsu et al*.* showed that serum IL-8 decreases after atezolizumab monotherapy in a cohort of more than 250 NSCLC patients. In particular, the lowest IL-8-fold change correlates with both a better PFS and objective response rate [266]. The variation in IL-8 serum levels represents an early efficacy biomarker for ICI response in NSCLC patients. Sanmamed et al*.* demonstrated that IL-8 serum levels decrease in responder NSCLC patients, whereas IL-8 upregulation is observed during progression in non-responder patients [267]. Patients with high baseline IL-8 plasma levels display shorter PFS and OS under ICI treatment [268]. Consistently, our recent meta-analysis confirms the predictive role of high IL-8 serum levels in lung cancer, especially in response to chemotherapy and immunotherapy [255]. In our meta-analysis on kidney cancer, a strong association between high circulating IL-8 levels and worse OS is observed primarily in patients treated with immunotherapy and, to a lesser extent, with the mTOR inhibitor everolimus and tyrosine kinase inhibitors (TKI) [256]. The predictive importance of IL-8 in RCC is also confirmed by Sharma et al*.*, who reported that IL-8 is elevated in patients resistant to targeted therapy (i.e., VEGF-TKI), thus highlighting the interest in targeting the IL-8/CXCR1/2 axis, to revert the resistance to VEGF inhibitors [269]. In large randomized studies conducted in advanced RCC and urothelial carcinoma patients, low IL-8 plasma levels are predictive for atezolizumab response. IL-8 is mainly expressed in both circulating and tumor-infiltrating myeloid cells: this in turn correlates with a decrease in antigen presentation [164]. IL-8 is also selectively released by papillary-non clear cell RCC (p-nccRCC) cells, as reported by Krawczyk et al*.* in preclinical models: together with chemerin and CXCL16, IL-8 is responsible for promoting not only monocyte recruitment but also their differentiation into foam-cell macrophages [270]. IL-8 levels also influence response to immuno- and targeted-therapy in melanoma. Elevated baseline IL-8 serum levels are linked to worse OS in response to paclitaxel and ipilimumab. At the same time, a decrease in IL-8 serum levels after ipilimumab therapy predicted objective response [243, 271]. This correlation is also observed for OS and PFS after BRAF and MEK targeted therapy [272]. Analysis of the CheckMate 067 trial comparing nivolumab, ipilimumab, or both in advanced melanoma, showed that IL-8 serum levels do not correlate with tumor PD-L1 expression, yet very effectively discriminate melanoma patients with better (low IL-8) or worse (high IL-8) OS, in all three immunotherapy treatment groups [273]. Circulating IL-8 levels correlate with *IL-8* gene expression by the tumor and blood neutrophil and monocyte counts and are negatively associated with tumor IFN-γ- and T-cell infiltration-related transcriptomic signatures and increased tumor infiltration by MPO^+^ and/or CD15^+^ monocytes and neutrophils. Collectively these data support the notion that tumor-produced IL-8 effectively shapes an immune suppressive TiME in melanoma, thereby predicting resistance to ICI.

#### Prognostic/predictive role of IL-8 polymorphisms

An overview of IL-8 SNP as a negative prognostic/predictive tool is reported in Table 2. As highlighted above, non‑coding IL-8 variants (SNP) have been associated with altered IL‑8 expression and different susceptibility or outcome in inflammatory, infectious, and neoplastic diseases. The -251 polymorphism with A allele (AA/AT) in the *IL-8* gene appears to increase the risk of developing GC; within the same genotype, cancer patients display increased IL-8 serum levels, as compared to healthy subjects [221]. In that respect, genotype/environment interactions explain how infection with *Helicobacter pylori*, one of the main risk factors in the development of GC, is associated with high serum levels of IL-8 and oxidative species [274]. Compelling evidence also associates higher risk of CRC to defined *IL-8* genotypes, such as the -251 T/A SNP [275]. Similar results, showing that SNP from genes within the IL-8 pathway (*IL-8*, *CXCR1* and *CXCR2*) are significantly associated with both colon and rectal cancer risk and an increased hazard ratio for death for patients affected by CRC, were also obtained by Bondurant et al*.* [149]. Since 2005, it has been shown that the *IL-8* - 251 T/A polymorphism is not associated with increased risk to develop lung cancer [276]. Such results were confirmed by a meta-analysis in 2015, showing that this SNP associates with increased risks to develop cancer only in Asian populations [277]. *IL-8* SNP may also be associated with patients’ outcome. A recent analysis in silico revealed that *IL-8* is one of three prognostic genes among 101 cancer-related genes in two public datasets of ESCA [278]. The *IL-8* - 251 T > A (A/A) SNP significantly correlates with unfavorable prognosis in GC patients, with a decreased time to tumor recurrence and OS [279]. Lurjie et al*.* demonstrated that CRC patients with *IL-8* - 251 A/A genotype are at greatest risk to develop tumor recurrence as compared to *IL-8* - 251 T/T patients [280]. Moreover, *IL-8* SNP not only display prognostic discrimination, but also potential predictive value regarding response to bevacizumab: indeed, Di Salvatore et al*.* showed reduced progression free survival (PFS) and OS in CRC patients treated with bevacizumab carrying -251 T/A or A/A SNP, as compared to those carrying TT alleles [281]. Evidence also supports the importance of *IL-8* polymorphisms in HCC progression. Patients harboring the *IL-8* -251 A/A variant recur early after surgery with shorter recurrence free survival, as compared to those harboring T allele variants (20 *vs* 32 months, respectively) [282]. Intrahepatic CCA (iCCA) patients harboring the *IL-8* -251 T/A or A/A genotype display shorter OS after surgery [283]. In perihilar CCA, the *CXCR1* + 860 C > G SNP significantly correlates with postoperative complications [284]. IL-8 SNP are associated with worse survival outcomes for targeted therapy also in advanced RCC patients treated with pazopanib and sunitinib [285].

## Therapeutic targeting of the IL-8/CXCR1/2 axis

Since IL-8 is a crucial mediator of cell communication into non-malignant diseases and TiME, its direct or indirect targeting for therapeutic purposes has become the focus of translational and early clinical research (Table 3) [129].

**Table 3:** Overview of the most characterized direct and indirect IL-8 therapeutics

Targeting	Type of strategy	Molecule	Mechanism of action	Preclinical evidence	Clinical trial	Ref
Direct	IL-8 neutralizing antibodies	HuMax-IL8 (BMS-986253)	Fully human IgG1κ antibody	Reduces the recruitment of PMN-MDSC and tumor growth in TNBC	NCT03689699; NCT04050462	[286, 287]
		ABX-IL8	Human IgG2 monoclonal antibody	Reduces tumor growth and metastasis; enhances innate immune response		[288, 289]
	CXCR1/2 inhibitors	Reparixin	Non-competitive allosteric inhibitor	Reduces cell viability and tumor growth; promotes antitumor immunity	NCT02001974	[170, 290–293]
		SB225002	Selective CXCR2 antagonist	Reduces cell proliferation; blocks neutrophil infiltration		[294–299]
		AZD5069	CXCR2 antagonist	Abolishes MDSC infiltration and activity		[262]
		Navarixin (SCH527123)	CXCR2 inhibitor	Reduces cell proliferation		[300]
		SB-656933	CXCR2 inhibitor	Reduces neutrophil accumulation	NCT00748410	
		SX-682	CXCR1/2 inhibitor	Inhibition of MDSC trafficking	NCT03161431, NCT05570825	[301–303]
Indirect	Targeting molecular cascades involved in IL-8 expression/production	Dabrafenib	BRAF inhibitor	Reduces/induces IL-8 transcription according to *BRAF* status		[23]
		Trametinib/PD0325901	MEK inhibitors	Inhibits IL-8 transcription		[23, 304]
			Knockdown of PTEN expression	Increased IL-8 expression		[41, 305]

Direct IL-8 axis inhibitors involve IL-8 neutralizing antibodies and CXCR1/2 inhibitors. The anti-IL-8 monoclonal HuMax-IL8, also known as BMS-986253, is a fully human IgG1κ antibody able to bind free IL-8, first tested in patients affected by palmoplantar pustulosis, a chronic inflammatory skin disease [306]. ABX-IL8 is a human IgG2 monoclonal antibody able to block the binding of IL-8 to its receptors [307]. Several therapeutic agents blocking IL-8 receptors have been developed [308]. Reparixin is a non-competitive allosteric inhibitor of CXCR1/2 and it showed therapeutic effects in both pre-clinical and clinical tests [309]. Specific CXCR2 antagonists are: SB225002 (selectivity maintained up to 3.3 μM), with anti-tubulin activity; AZD5069 (pIC_50_ = 9.1); navarixin (also known as SCH527123) (IC_50_ = 2.6 nM) [295, 308, 310–312]. As IL-8 expression is regulated at multiple levels (see above), the IL-8 axis can also be modulated by targeting other molecular cascades (e.g., MAPK and PI3K).

### Clinical applications in non-malignant diseases

Collectively, IL-8-targeting therapeutics may limit neutrophil trafficking and reduce NET burden and downstream inflammatory amplification, positioning this pathway as an attractive strategy to modulate neutrophil-driven pathology, especially in UC [313]. In UC mouse models, network analysis showed that IL-8 and its receptors are the main target of quercetin. Quercetin effectively reduces IL-8 and its receptors, in parallel with restoration of colonic crypt architecture and reduction of inflammatory cell infiltration [314]. Preclinical in vivo experiments demonstrated that the administration of G31P, a mutant isoform of IL-8 acting as a CXCR1/2 antagonist, restrains macrophage infiltration and enhances tight junction proteins [315]. The CXCR2 inhibitor SB225002 also improves clinical outcome, by reducing neutrophil recruitment in colitis mice models [299]. Building on this preclinical evidence, clinical trials targeting CXCR1/2 have been developed. For example, the CXCR2 inhibitor SB-656933 has been investigated in UC due to its ability to reduce neutrophil accumulation (NCT00748410). Another trial is recruiting patients with UC to determine the safety and efficacy of eltrekibart, an anti-CXCR2 ligands monoclonal antibody, and mirikizumab, a monoclonal antibody approved for UC treatment (NCT06598943).

Current evidence suggests that targeting IL-8 or its receptors, especially with CXCR2 antagonists, may be promising also in preclinical studies of neuroinflammatory and neurodegenerative diseases, but real-world applications remain limited [316]. In an animal model of Alzheimer's disease, blocking CXCR2 with SB332235 reduces microgliosis, oxidative stress and neuronal loss. This shows neuroprotective effects and limits inflammatory responses after amyloid-β challenges [317]. Antibody-based methods targeting CXCR2 have also been effective at blocking IL-8-driven neutrophils’ migration and alleviating symptoms in experimental autoimmune encephalomyelitis, highlighting the potential of receptor blockade in neuroinflammatory conditions [318]. IL-8/CXCR2 antagonists are currently under investigation for a range of inflammatory and neurodegenerative disorders, and nonsteroidal anti-inflammatory drugs, such as ibuprofen, suppress IL-8-driven neuroimmune dysregulation in cross-disease molecular analyses [319, 320]. However, clinical trials in neuroinflammatory diseases have shown mixed results, with ongoing challenges in BBB penetration, unwanted side effects, and patient selection [321, 322]. Further studies are needed to better understand the underlying mechanisms, optimize dosing strategies, and improve CNS delivery.

While clinical studies consistently support IL-8 as a robust prognostic marker in diverse cardiovascular settings, experimental data also suggest biologically active roles beyond passive association, highlighting IL-8/CXCR1/2 pathways as potential targets for future anti-inflammatory and vascular-protective strategies. For example, it has been demonstrated that the CXCR1/2 inhibitor reparixin decreases systolic blood pressure and increased the blood flow in rats [323]. Also the CXCR2 inhibitor SB225002 reduces elevation of blood pressure and cardiac myocyte hypertrophy in hypertensive rat models [324].

### Applications in oncology

In preclinical models of triple-negative breast cancer (TNBC), HuMax-IL8 reduces the recruitment of PMN-MDSC and enhances the susceptibility of claudin-low breast cancer cells to immune-mediated lysis [287]. Song et al*.* recently demonstrated the synergistic effects of HuMax-IL8 *plus* anti-PD-L1 antibody (atezolizumab) in reducing tumor growth in mouse models of TNBC [286]. A phase I trial showed that HuMax-IL8 is safe and well-tolerated in patients with metastatic or unresectable solid tumors [325]. A novel bispecific antibody (BP2402) targeting both pathways has been shown to exert significant antitumor effects in vivo [286]. Several studies are investigating the combination of IL-8 blockade and ICI, for example in hormone-sensitive prostate cancer (NCT03689699) and advanced HCC (NCT04050462). Huang et al*.* showed that ABX-IL8 reduces tumor growth and metastasis in murine melanoma models [288]. A preclinical study by Li et al*.* demonstrated the efficacy of the combination of an anti-IL-8 antibody with immunotherapy: the use of the anti-IL-8 antibody re-programs myeloid cells, enhancing the innate immune response and antitumoral activity of anti-PD-1 in a humanized murine model of pancreatic cancer [289].

Regarding the IL-8 receptors inhibitors, reparixin reduces cell viability of CXCR1^+^ breast cancer cells and tumor growth in combination with docetaxel in human breast cancer xenografts [290]. In GC, reparixin decreases PD-L1^+^ macrophages, promoting antitumor immunity [170]. An in vitro study showed that reparixin also increases the chemotherapeutic efficacy of 5-fluorouracil in GC cells by increasing apoptosis, as well as inhibiting cell migration and invasion [291]. Reparixin treatment reduces cell proliferation, EMT and stemness also in in vitro models of human epithelial thyroid cancer [292]. On the contrary, our preclinical experiments with CRC models showed that reparixin does not impact cell viability [294]. Although a phase Ib pilot study with the combination of reparixin *plus* paclitaxel in HER-2^−^ breast cancer patients demonstrated objective responses to treatment this agent does not appear to have progressed to later phase clinical trials [293]. We and others have demonstrated that the selective CXCR2 antagonist SB225002 strongly reduces cell proliferation, by inducing a G2/M phase cell cycle block and apoptotic cell death in CRC cell lines [294, 295]. These anti-proliferative effects of SB225002 have been validated in several cancers of different histological origin, like prostate, cervical and lung [296–298]. Importantly, we also found that SB225002 arrests cell proliferation of fibroblasts and endothelial cells, suggesting the relevance of this drug also in impacting the surrounding TiME [294]. Consistently, Ni et al*.* demonstrated that SB225002 enhances radiotherapy efficacy by blocking CXCR2-dependent neutrophil infiltration in cervical cancer [297]. Sueoka et al*.* showed that patients with higher levels of CXCR2 show poor prognosis, and proliferation, invasion and migration are significantly reduced by CXCR2 inhibition by either SB225002 or CXCR2 siRNA [326]. Tumor derived-IL-8 activates NF-κB, inducing a positive feedback loop that increases tumor aggressiveness and stemness in preclinical models of iCCA. This effect is reversed by the specific CXCR2-inhibitor SB225002 [327]. Targeting CXCR2 with its antagonist AZD5069 abolishes MDSC infiltration and activity, thus restoring the response to anti-PD-L1 [262]. SCH527123 shows antiproliferative effects in in vitro CRC models, suppressing the CXCR2-mediated molecular pathways, such as PI3K and MAPK [300]. However, none of these CXCR2-targeting agents appears to be in active clinical development at present. SX-682, a small-molecule inhibitor of both CXCR1 and CXCR2, has been observed as a therapeutic advantage in immunotherapy regimen. For example, the phase I clinical trial combining SX-682 with pembrolizumab in melanoma patients, not only shows tolerable safety profile but also disease control during progression (NCT03161431). In NSCLC, the same combination schedule in a phase II clinical trial increases the effects of immunotherapy, as compared to single administration (NCT05570825).

Our group demonstrated that nuclear translocation of the CHOP transcription factor, controlled by activation of the MAPK pathway, leads to *IL-8* transcription. The MEK inhibitor trametinib decreases IL-8 production by retaining CHOP into the cytoplasm; conversely, the BRAF inhibitor dabrafenib behaves similarly to trametinib in *BRAF*-mutant CRC cells. In *BRAF*-wt contexts, dabrafenib induces paradoxical MAPK activation, CHOP nuclear translocation, and IL-8 production [23]. Another MEK inhibitor, PD0325901, inhibits *IL-8* transcription in melanoma cell lines regardless of *BRAF* status [328]. In melanoma and glioblastoma models in vitro, genetic manipulation of the anti-apoptotic protein Bcl-XL affects *IL-8* transcription [304]. Along with the MAPK pathway, PI3K signaling is another driver regulator for IL-8 production. Maxwell et al*.* found that the knockdown of PTEN expression correlates with increased IL-8 expression in prostate cancer cells [41]. This evidence has been also observed in HNSCC cell lines, suggesting a broad molecular mechanism [305]. Overall, such evidence suggests that inhibitors of the MAPK and PI3K pathways may exert their anti-cancer effects, at least in part, by modulating IL-8 production and therefore potentially contribute to therapeutic strategies aimed at indirectly modulating the IL-8/CXCR1/2 axis.

### Current challenges and future perspectives for targeting IL-8/CXCR1/2 Axis

Despite the increasing number of the therapeutic targeting of the IL-8/CXCR1/2 axis described, there are not many clinical studies in both oncology and non-oncological diseases that can actually lead to their clinical application. Multiple reasons can explain this lack in the clinical practice. As we above discussed, the first point can be ascribed to the lack of the standardization of the method/source of IL-8 detection [207]. IL-8 mAb and CXCR2 specific inhibitors lead to the increase of other ELR^+^ CXCL ligand that can rescue the activation of the downstream pathway [329]. Moreover, by inhibiting CXCR1 or CXCR2, these inhibitors induce compensatory alternative molecular cascades [330]. Overall, these molecular rescues render the upstream inhibition limited. The last critical point is related to the experimental models used for characterizing the IL-8/CXCR1/2 inhibitors effects in tissue microenvironment, as they cannot be related to preclinical 2D models. In that respect, the interactions with the elements of immune system require complex models, like mice, which unfortunately lack of a homologue of human *IL-8* [331]. Collectively, these issues have to be considered to design new clinical trials in the next future to fully established new IL-8-based therapeutic options.

## Conclusion

**Fig. 3:**
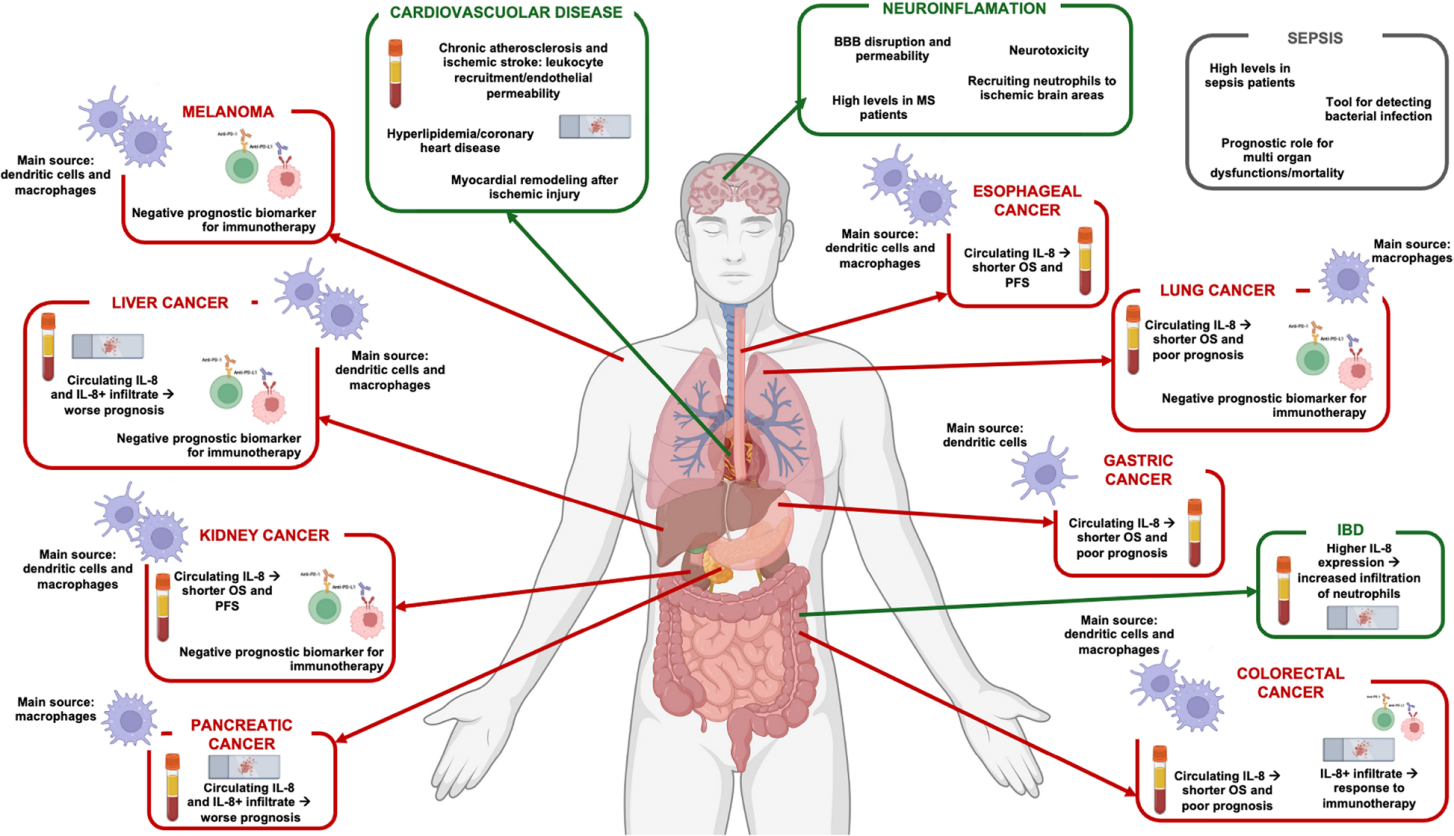
IL-8 functions in health and disease. Overview of IL-8 implications in non-malignant diseases (green arrows) and solid cancers (red arrows). According to the type of tumor, it is highlighted the involvement of IL-8 in immunotherapy response and the main immune cells source

The incidence and the mortality of multiple severe immunological disorders, including sepsis, cardiovascular and neurological disease, atherosclerosis and cancer remain dramatic. In this scenario, multiple lines of evidence support an important role of the IL-8 chemokine and its receptors CXCR1/2, due to their involvement in key pathophysiological processes, such as response to infections, inflammation, tissue remodeling, immune response and cancer (Fig. 3). In particular, IL-8 may serve as a prognostic and predictive biomarker, whose detection may impact treatment algorithms in clinical practice. Moreover, many IL-8/CXCR1/2-targeting agents have been developed and tested. In cancer, IL-8 can be used as an innovative prognostic biomarker and, potentially, as a predictor of response to cancer immunotherapy.

Data discussed here clearly highlight that IL-8 can act as both friend or foe according to the histotype (e.g., CRC or RCC) and the detection (e.g., circulating or tissue). Hence, the current clinical need is related to the standardization of the source (i.e., plasma or tissue) and methods (i.e., polymorphisms, mRNA or protein) of IL-8 assessment in diverse clinical settings to fully establish and clinically validate IL-8 as a novel biomarker.

## Data Availability

Not applicable.
